# Erratum

**DOI:** 10.1590/0037-8682-201200342013

**Published:** 2020-12-18

**Revista da Sociedade Brasileira de Medicina Tropical/*Journal of the Brazilian Society of Tropical Medicine***

**Title:** *Anopheles deaneorum:* a new potential malaria vector in State of Santa Catarina, Brazil (Diptera: Culicidae) 

**Vol.:46(1): 2013** - doi: https://doi.org/10.1590/0037-8682201200342013 - AUTHOR

**Page 121**

**Vinicios Ferreira de Freitas**

Should read:

**Lílian Ferreira de Freitas**

